# Biomechanics of Double Poling in Paralympic Cross-Country Skiing—A Cross-Sectional Study Comparing the Standing and Sitting Positions in Healthy Male Subjects

**DOI:** 10.3390/medicina58020201

**Published:** 2022-01-28

**Authors:** Junpei Sasadai, Noriaki Maeda, Masanori Morikawa, Makoto Komiya, Reia Shimizu, Kazuki Fukui, Mitsuhiro Yoshimi, Yoshifumi Kono, Yukio Urabe

**Affiliations:** 1 Sports Medical Center, Japan Institute of Sports Sciences, Tokyo 115-0056, Japan; reia.shimizu@jpnsport.go.jp; 2 Graduate School of Biomedical and Health Sciences, Hiroshima University, Hiroshima 734-8553, Japan; makoto-komiya@hiroshima-u.ac.jp (M.K.); kazuki-fukui@hiroshima-u.ac.jp (K.F.); mitsuhiroyoshimi0116@hiroshima-u.ac.jp (M.Y.); yurabe@hiroshima-u.ac.jp (Y.U.); 3 Center for Gerontology and Social Science, National Center for Geriatrics and Gerontology, Aichi 474-8511, Japan; morikawa@ncgg.go.jp; 4 Division of Rehabilitation, Department of Clinical Practice and Support, Hiroshima University Hospital, Hiroshima University, Hiroshima 734-8551, Japan; ykono118@hiroshima-u.ac.jp

**Keywords:** cross-country skiing, double poling, biomechanics, Paralympics, athletes with disabilities

## Abstract

*Background and Objectives*: Double poling is an important fundamental skill required for cross-country skiing in able-bodied athletes and in those with physical disabilities. Meanwhile, the performance improvement and injury prevention related to double poling requires a thorough assessment, whereas the scapular and shoulder kinematics in different postural conditions remain to be clarified. The main purpose of this study was to evaluate the biomechanics during cross-country ski double poling in the standing and sitting positions. *Materials and Methods*: Eleven participants underwent kinematic assessments of the shoulder girdle during double poling on a ski ergometer with an electromagnetic tracking device. The cycle rate, stroke length, stroke speed, thorax motion relative to pelvis, scapular motions relative to thorax, humeral motions relative to thorax, and humeral motions relative to scapula were calculated for five double-poling cycles. *Results*: In the sitting position, the angles of humerothoracic elevation were 18 degrees larger and glenohumeral elevation 13 degrees larger than in the standing position at the upward point and range of motion. *Conclusions*: The study revealed that double poling in the sitting condition increased the humerothoracic and glenohumeral elevation angle to secure the poling margin. If these are excessive, there is a risk of shoulder injuries such as subacromial impingement.

## 1. Introduction

Paralympic cross-country skiing has been listed as an official sport of the Paralympic Games since 1976 [1]. Since the first Paralympic event, the number of athletes practicing cross-country skiing has risen markedly [1]. Double poling is among the most important and fundamental skills required for cross-country ski racing, wherein the goal is to complete the set distance as fast as possible [2]. Until now, researchers have examined several performance-related aspects of double poling, including the biomechanics or the physical function, for able-bodied athletes [3]. Recently, studies including skiers with physical disabilities have been performed [4,5]. Double-poling requires holding the ski poles with the hands; hence, some studies emphasized the contribution of upper-limbs during double poling [3,6]. One recent study mentioned the scapular movement during double poling [7], but did not analyze the biomechanics in detail. In parallel, other studies described the influence of trunk and lower limb motion during double poling [8,9]. Holmberg et al. [10] reported that the knee and ankle joint movements are integral to performing the double-poling technique, and movement restrictions in these joints significantly affects the biomechanical as well as the physiological variables, which lead to an impaired double-poling performance. Therefore, coordinating the motions of upper limbs, trunk, pelvis, and lower limbs is considered vital during double poling. We assumed that a para cross-country skier with any degree of impairment in the lower limb, pelvis, or trunk may require a higher contribution of the upper limbs during poling than that required by able-bodied athletes. However, there are few studies comparing the differences between standing and seated double poling on the body. In a previous study, Tervo et al. [11] reported that quadriceps muscle activity increased as the intensity of the double pole increased in the standing position, whereas there was no change in the seated position. This study assessed the difference in stand-up athlete’s muscle activity of the rectus femoris in standing and sitting using a double-pole ergometer. The differences in the biomechanics of the upper extremities that actually apply the force to the poles have not been elucidated until now.

In able-bodied cross-country skiing, lower back pain is common, which is believed to occur because of the specific loading patterns of skiing, such as double poling [12]. However, approximately 50% of the total injuries in para cross-country skiing were reported to occur in the upper extremities and almost half of them involved the shoulder [13]. Ohlsson et al. revealed that the range of the resultant reaction force in the shoulder joint was nearly thrice the body mass, which may cause those shoulder injuries in able-bodied sit-ski athletes [14]. Thus, an understanding of the kinematics of the upper extremity, especially scapular and shoulder during double-poling is not only necessary for improving performance, but may also be useful for preventing injury. However, very few studies presented a comparison of the poling characteristics between the standing and sitting positions [11], and this has resulted in a lack of clarity regarding the scapular and shoulder kinematics during double poling with different positions (standing and sitting). 

The purpose of this study was to clarify the biomechanical differences, especially scapular and shoulder kinematics, during cross-country ski double poling between standing and sitting with healthy individuals.

## 2. Materials and Methods

### 2.1. Participants

We included 11 healthy male recreational skiers (mean age: 24.7 ± 3.1 years; height: 170.9 ± 5.3 cm; body mass: 63.9 ± 10.0 kg; BMI: 21.8 ± 2.6 kg/m^2^). The inclusion criteria were that the subjects had skied regularly several times a year and had mastered the technique of double poling. The technique was judged by one skilled ski instructor. Subjects with any history of shoulder injuries in ≥12 months before the day of measurement were excluded. 

This study was approved by the epidemiologic study ethics review board of Hiroshima University (approval number: E-1365), and all participants provided written informed consent before participating in this study. This investigation was conducted according to the principles expressed in the Declaration of Helsinki, 7th edition.

### 2.2. Design

Our study had a cross-sectional design. Each position was considered as an independent variable, and the kinematic variables of double poling were considered as dependent variables. Participants attended a 1 h testing session at the university and were tested in a single session while remaining in the standing and sitting positions in a randomized order.

### 2.3. Measurement

**Figure 1:**
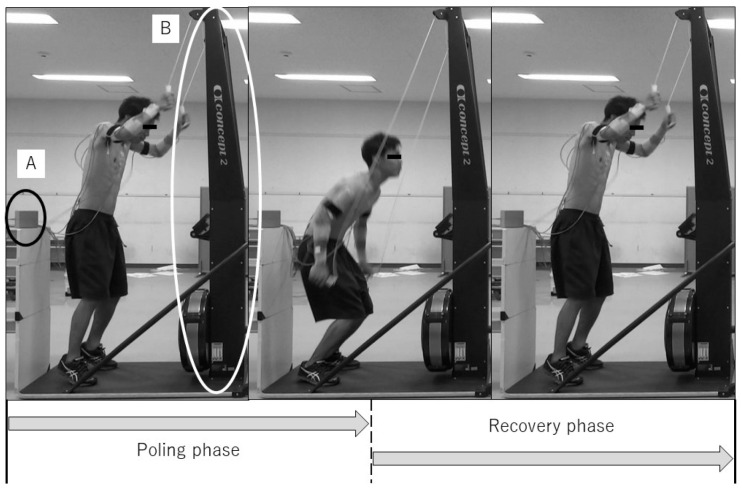
Measurement environment and phase division. (**A**) the transmitter of the electromagnetic tracking device; (**B**) the ski ergometer.

Using an electromagnetic tracking device with a system electronics unit, transmitter, and eight sensors (Liberty, Polhemus, Colchester, VT, USA), the kinematic measurements were taken for the participants during double poling on a ski ergometer (Skierg, Concept2^®^, Morrisville, VT, USA) that has generally measured double poling in cross-country skiers [5,6,15]. Participants were assigned to two randomly ordered experimental conditions (standing and sitting positions), and they performed three trials of 15 consecutive maximal-effort double poling. In each condition, a 5 min warm-up with ski ergometer was performed before the measurements. To avoid fatigue, recovery between trials was set to 3 min. A 40 cm chair without a backrest not affecting the magnetic field was used for the trials in the sitting position. The transmitter defining the global coordinate system was placed near the participant. The sensors were attached on the participant’s sternum, sacrum, and the acromion, humerus, and forearm bilaterally. A six-body segment model emulating the pelvis, thorax, scapula, and humerus was developed to detect the 3D motion of the trunk and arm. Each segment was defined by the digitized body landmarks based on the definition of a joint coordinate system proposed by the International Society of Biomechanics [16]. The kinematic data during double poling were collected at a sampling frequency of 240 Hz and the side of the limb to be assessed was selected as their dominant side. The cycle rate of poling was defined by the location of the sensor attached to the distal forearm; the cycle of poling was classified into the poling phase and the recovery phase (Figure 1). The poling phase was defined as the period from when the sensor was attached to the forearm located at the most upward (Upward) point to the point when that reached the most downward (Downward) point. In contrast, the recovery phase was defined as the period from the Downward to the next Upward. The stroke length and stroke speed were calculated for each phase. The stroke length was calculated based on the location of the sensor attached on the forearm. The stroke speed was calculated by dividing the poling length by the time for each phase. Additionally, the stroke rate of each cycle was calculated using the time required for the sensor attached to the forearm to perform one cycle from the time-point when participants started poling. Regarding the kinematic data, the scapular motion relative to thorax (protraction, upward rotation, and posterior tilt), the humeral motion relative to the thorax (denoted by the plane of elevation that indicates horizontal adduction, elevation, and internal rotation), and the humeral motion relative to the scapula (denoted by the plane of elevation indicating horizontal adduction, elevation, and internal rotation) at the two time points (Upward and Downward) in each cycle. Additionally, the range of motion (ROM) for each biomechanical parameter was calculated as the difference between the values at the Upward and Downward points. Mean data of the six to tenth cycle of double poling was analyzed in this study.

### 2.4. Statistical Analysis

Continuous data were expressed as means and standard deviation (SD). The normal distribution of each variable was confirmed using the Shapiro–Wilk test. The differences in the parametric variables between the groups were determined with a Student’s *t*-test with paired samples. For non-parametric variables, the differences were determined by Wilcoxon’s signed-rank test. An α level of 0.05 was the criterion for rejection of the null hypothesis for all statistical tests. Statistical analysis was conducted on SPSS for Windows, version 23.0 (IBM Japan Co., Tokyo, Japan).

## 3. Results

### 3.1. Variables of Double-Poling Performance

As shown in Table 1, all variables of double-poling performance except the average stroke rate were significantly different between the standing and sitting positions. The stroke length of the poling phase while double poling in the sitting position was significantly shorter than that of the standing position (*p* = 0.036). The stroke speed of the poling phase in the sitting position was significantly slower than that recorded in the standing position in each cycle (*p* = 0.004). Also, the stroke length of the double poling was significantly shorter in the recovery phase in the sitting position than that in the standing (*p* = 0.045) position. The stroke speed of the recovery phase in the sitting position was significantly lesser than that while standing in each cycle (*p* = 0.026). The power output of poling in the sitting positions was significantly smaller than that while standing (*p* < 0.001). The stroke rates were not significantly different between the two positions (*p* = 0.275).

### 3.2. Kinematics of Double Poling

Table 2 shows the kinematic data of the upper-limb during poling phase compared between the standing and sitting positions while double poling. Although the scapulothoracic motions in standing and sitting positions were not significantly different, the upward rotation angle tended to be larger for participants in the sitting condition at the Upward (*p* = 0.083) and Downward (*p* = 0.062) points.

The angle of humerothoracic elevation at the Upward (*p* = 0.006) point and the ROM of humerothoracic elevation (*p* = 0.006) showed significant differences in the data recorded for the standing and sitting positions, respectively. Furthermore, the angle of humerothoracic internal rotation at the Upward (*p* = 0.037) and Downward (*p* = 0.030) points were significantly different between the standing and sitting positions.

The angle of glenohumeral plane of elevation was smaller in the sitting position than that in the standing condition (*p* = 0.004); the angle of glenohumeral elevation at the Upward point was significantly larger in the sitting position than that in the standing condition (*p* = 0.001). In contrast, the ROM of glenohumeral elevation was larger in the sitting condition (*p* = 0.006). There were no significant differences in the glenohumeral internal rotation between both postural conditions.

Table 3 presents the kinematic data of the thoraco-pelvic ROM. Thoracopelvic flexion showed no difference between the postural conditions at the Upward point, while it was significantly larger in the sitting condition at the Downward point (*p* < 0.001). There was also a significant difference of ROM between the two postural conditions (*p* = 0.018). The thoracopelvic lateral flexion showed significant difference between standing and sitting in the Upward (*p* = 0.001), Downward (*p* = 0.009) and ROM (*p* = 0.017). There was no significant difference in the thoracopelvic rotation between both postural conditions.

## 4. Discussion

This study aimed to clarify the differences in cross-country ski biomechanics while double poling in the standing and sitting positions in healthy men. Therefore, parameters such as the stroke rate, stroke length, stroke speed, and the kinematics of scapular, shoulder and trunk during double poling were measured and analyzed. The analysis of these performance-related variables of the kinematics during double poling demonstrated that the participants had a lower performance while sit-skiing than that of the participants while skiing in the standing position. The kinematic data revealed that there was a difference in the upper limb and trunk movement with the scapula while sitting and standing. This is the first study to analyze the scapular and shoulder joint movements in the double-poling motion in the standing and sitting positions for healthy individuals.

Although the cycle rate was similar between the two positions, the stroke length and speed of the poling and recovery phases were larger for the healthy skiers in the standing position. This indicates that the participants performed double poling faster and longer in the standing position than in the sitting position. The comparison between the high-level and the regional-level able-bodied skiers demonstrated no difference in the time of the poling phase [17]. Regarding double poling in the standing condition in this study, high-level skiers were speculated to complete longer lengths at higher speeds while double poling in both positions. In fact, the power outputs were 1.56-times higher when able-bodied skiers double poled in the standing position than in the sitting position. In a previous study, Holmberg et al. [10] reported that the force applied to a double pole with the ankle and knee joints restricted by braces was 10.9% lower than that without restrictions. In the sitting position, the lower extremities are similarly not available for double poling. Therefore, our results suggested that the double poling performance in the sitting position was below that with the standing position.

From the kinematic analysis, the humerothoracic angle, which measures the movement of the shoulder complex, showed a difference in the elevation between standing and sitting. This suggested that in the sitting condition, the poling margin is secured by increasing the shoulder complex elevation angle Upward, although the stroke length is shorter than that while standing.

In the Paralympic cross-country skiing competitions, sit-ski athletes often prefer long poles, thus a similar trend in participants with no sit ski experience may indicate the validity of this study. Stöggl et al. [9] reported that an elite able-bodied athlete had a shoulder elevation angle of 79 to 84° when touching the pole in the optical motion analysis. This result has a difference of about 9° from the measurement recorded in our study for double poling in the standing position, which is reported to be influenced by the difference between the actual ski pole and ski ergometer or the different phase regulations. Conversely, the humerothoracic elevation angle while double poling in the sitting position was 116°, which was almost similar to that reported previously study on professional sit-ski athletes [18]. From these analyses, it was confirmed that a large elevation angle is required for double poling in sit skiing.

Although some tendency was observed in the upward rotation of the scapula (34.78° vs. 40.99°), the scapulothoracic angles were not statistically significant between standing and sitting; the scapular movement does not change with the standing or sitting conditions in the healthy able-bodied participants. The humeral elevation angle is a combination of movements of scapulothoracic and glenohumeral joint, as described by Codman as the “scapulohumeral rhythm” [19]. In recent years, it has become clear that the scapulohumeral rhythm is not constant but variable, depending on differences in movement and individual differences [20,21]. Therefore, the difference in the humerothoracic elevation angle of about 20° at the Upward point may be related to the movement of the glenohumeral joint. Our results confirmed that the glenohumeral elevation angle is larger while sitting than while standing at the Upward point. Moreover, the glenohumeral plane of elevation was significantly lower while sit-skiing, suggesting that there is more elevation in the abduction direction while sitting than that during standing and double-poling. Generally, the risk of subacromial impingement increases when an internal rotation is added to the elevation angle [22,23]. These results show that although there is no excess glenohumeral internal rotation, the humerothoracic angle showed significant internal rotation at both the Upward and Downward points in the sitting condition. Increases in the elevation angle of the glenohumeral joint and internal rotation of the humerothoracic angle on sit-ski may cause subacromial impingement. The decreased mobility of the scapula and strength of the shoulder girdle are involved in shoulder injuries, and influence the sports performance as well [24,25]; however, shoulder injuries may occur by the same mechanism in sit-ski athletes.

When focusing on the thoraco-pelvic angle, a larger flexion angle was observed for the sitting position than for the standing position at the Downward point and ROM. Trunk flexion is another important force, which is crucial when the lower limbs cannot be used in the sitting position. Gastaldi et al. [18] also reported that trunk flexion allows the shoulder and elbow not to be strained, and for generating a greater force and limiting fatigue. The results of this study also indicate that trunk mobility and strength may be important in double poling for athletes with residual trunk muscle strength. Furthermore, in this study, measurements were taken in a chair sitting position with the hip and knee joints flexed approximately 90°, but in actual sit-skiing, various types such as long sitting and kneeling are chosen by the athletes according to their physical functions. In a previous study in which double-poling movements of sit-skiers with different disability classes were analyzed in 2D, several variations were observed in sitting and whole-body joint movements in the sagittal plane [18]. It is possible that functions such as flexibility and muscle strength of the lower limbs, which were not investigated in this study, may also affect trunk and upper limb movements as a kinetic chain.

We also noted that there were some limitations in our study. First, we analyzed only healthy male participants without any experience of Paralympic skiing competitions. The impairment of each sit skier varies; hence, the range of motion, muscle strength, or muscle endurance of each participant might have differed accordingly. A cohort including more sit-skiers would help in comparing between able-bodied athletes and para-athletes. Second, we measured the biomechanical parameters during double poling using a ski ergometer. The kinematics of poling on the ski ergometer may differ from that of actual poling using skies and poles described in a previous study [26]. Future studies regarding poling biomechanics using a ski or roller-ski may provide more knowledge on cross-country skiing athletes.

## 5. Conclusions

In this study, the kinematics data of scapular, shoulder and trunk kinematics during cross-country ski double poling were measured with an electromagnetic tracking device and the scapular and shoulder kinematics for the performance in the two postural positions (standing and sitting) were analyzed. This is the first study to analyze the shoulder and scapula biomechanics of double poling by comparing the standing and sitting positions. The study revealed that double poling in the sitting position increased the humerothoracic and glenohumeral elevation angles to secure the margin for poling. When these are excessive, there is a risk of shoulder injuries by subacromial impingement. Physical therapists, athletic trainers, and athletes should be aware of these implications, and should train athletes to improve their performance while preventing shoulder injuries. In order to prevent overloading the glenohumeral joint, it may be important to improve their residual function of trunk and lower extremity, and mobility of the thorax and scapula.

## Figures and Tables

**Table 1:** The variables of double poling performance in each position.

	Standing	Sitting	*p*-Value
Stroke rate (strokes/min)	63.99 ± 8.70	58.47 ± 12.12	0.275 ^a^
Stroke length of poling phase (cm)	**109.37 ± 12.46**	**99.59 ± 9.89**	**0.036 ^a^**
Stroke speed of poling phase (cm/s)	**213.93 ± 17.50**	**189.00 ± 15.78**	**0.004 ^a^**
Stroke length of recovery phase (cm)	**108.67 ± 13.11**	**99.17 ± 10.01**	**0.045 ^a^**
Stroke speed of recovery phase (cm/s)	**260.54 ± 60.54**	**231.69 ± 33.29**	**0.026 ^b^**
Power output (W)	**226.93 ± 47.71**	**145.20 ± 26.57**	**<0.001 ^a^**

The values are presented as mean ± standard deviation. ^a^ Paired student’s *t*-test, ^b^ Wilcoxon signed rank test. Bold letter: indicates a significant difference between standing and sitting (*p* < 0.05).

**Table 2:** The kinematic data of upper limbs during double poling in each position.

		Standing	Sitting	*p*-Value
Scapulothoracic Protraction (degree)	Upward	22.98 ± 10.18	27.48 ± 7.40	0.138 ^a^
	Downward	38.96 ± 7.46	40.23 ± 8.46	0.458 ^a^
	ROM	−15.98 ± 7.62	−12.76 ± 9.50	0.268 ^a^
Scapulothoracic Upward Rotation (degree)	Upward	34.78 ± 12.11	40.99 ± 5.21	0.083 ^a^
	Downward	6.45 ± 6.75	8.78 ± 3.95	0.062 ^b^
	ROM	28.32 ± 12.20	32.20 ± 5.25	0.212 ^a^
Scapulothoracic Posterior Tilt (degree)	Upward	11.68 ± 15.11	9.19 ± 17.53	0.366 ^a^
	Downward	−7.96 ± 10.72	−11.03 ± 6.05	0.150 ^a^
	ROM	19.64 ± 12.77	20.23 ± 18.09	0.929 ^b^
Humerothoracic Plane of Elevation (degree)	Upward	53.29 ± 12.45	52.87 ± 13.30	0.908 ^a^
	Downward	47.00 ± 13.93	39.56 ± 16.67	0.110 ^b^
	ROM	6.29 ± 21.21	13.30 ± 21.99	0.199 ^a^
Humerothoracic Elevation (degree)	Upward	**97.72 ± 11.48**	**116.14 ± 11.18**	**0.006 ^a^**
	Downward	30.98 ± 10.20	27.77 ± 5.26	0.353 ^a^
	ROM	**66.74 ± 18.33**	**88.37 ± 10.32**	**0.006 ^a^**
Humerothoracic Internal Rotation (degree)	Upward	**−58.45 ± 13.47**	**−51.80 ± 9.77**	**0.037 ^a^**
	Downward	**−26.80 ± 22.21**	**−16.16 ± 18.83**	**0.030 ^a^**
	ROM	−31.65 ± 18.00	−35.64 ± 17.18	0.282 ^a^
Glenohumeral Plane of Elevation (degree)	Upward	**28.40 ± 10.39**	**19.85 ± 9.56**	**0.004 ^a^**
	Downward	29.08 ± 10.36	27.41 ± 19.78	0.859 ^b^
	ROM	0.68 ± 6.50	−7.56 ± 20.56	0.184 ^a^
Glenohumeral Elevation (degree)	Upward	**62.17 ± 11.46**	**75.27 ± 12.38**	**0.001 ^a^**
	Downward	26.83 ± 11.06	21.99 ± 8.16	0.200 ^a^
	ROM	**−35.34 ± 7.89**	**53.27 ± 12.55**	**0.006 ^b^**
Glenohumeral Internal Rotation (degree)	Upward	−66.81 ± 17.53	−60.59 ± 18.44	0.098 ^a^
	Downward	−46.64 ± 25.99	−46.19 ± 22.21	0.961 ^a^
	ROM	−20.16 ± 20.49	−14.40 ± 29.34	0.578 ^a^

The values are presented as mean ± standard deviation. Upward: the time point when the sensor attached to forearm located at the most upward; Downward: the time point when the sensor attached to forearm located at the most downward; ROM: the difference between the value at Upward and that at Downward ([Upward]—[Downward]). ^a^ Paired Student’s *t*-test, ^b^ Wilcoxon signed rank test. Bold letter: indicates a significant difference between standing and sitting (*p* < 0.05).

**Table 3:** The kinematic data of thoracopelvis during double poling in each position.

		Standing	Sitting	*p*-Value
Thoracopelvic flexion (degree)	Upward	24.68 ± 12.57	22.50 ± 11.21	0.641 ^a^
	Downward	**52.90 ± 11.65**	**64.84 ± 10.05**	**<0.001 ^b^**
	ROM	**28.22 ± 13.96**	**42.34 ± 9.69**	**0.018 ^a^**
Thoracopelvic left lateral flexion (degree)	Upward	**−3.41 ± 5.48**	**1.48 ± 4.50**	**0.001 ^a^**
	Downward	**−0.51 ± 4.52**	**1.40 ± 3.88**	**0.009 ^a^**
	ROM	**−2.91 ± 2.74**	**0.07 ± 4.78**	**0.017 ^a^**
Thoracopelvic left rotation (degree)	Upward	4.88 ± 7.03	4.08 ± 5.38	0.363 ^a^
	Downward	1.80 ± 5.60	1.84 ± 7.21	0.657 ^b^
	ROM	3.08 ± 4.04	2.23 ± 3.69	0.605 ^a^

The values are presented as mean ± standard deviation. Upward: the time point when the sensor attached to forearm located at the most upward; Downward: the time point when the sensor attached to forearm located at the most downward; ROM: the difference between the value at Upward and that at Downward ([Upward]—[Downward]). ^a^ Paired Student’s *t*-test, ^b^ Wilcoxon signed rank test. Bold letter: indicates a significant difference between standing and sitting (*p* < 0.05).

## Data Availability

The data presented in this study are available on request from the corresponding author.
